# 2-Amino-5-chloro­pyrimidin-1-ium hydrogen maleate

**DOI:** 10.1107/S1600536811051646

**Published:** 2011-12-14

**Authors:** Hoong-Kun Fun, Madhukar Hemamalini, Venkatachalam Rajakannan

**Affiliations:** a X-ray Crystallography Unit, School of Physics, Universiti Sains Malaysia, 11800 USM, Penang, Malaysia; b Biomedical Structural Biology, School of Biological Sciences, Nanyang Technological University, Singapore 138673

## Abstract

In the title salt, C_4_H_5_ClN_3_^+^·C_4_H_3_O_4_^−^, the 2-amino-5-chloro­pyrimidinium cation is protonated at one of its pyrimidine N atoms. In the roughly planar (r.m.s. deviation = 0.026 Å) hydrogen malate anion, an intra­molecular O—H⋯O hydrogen bond generates an *S*(7) ring. In the crystal, the protonated N atom and the 2-amino group of the cation are hydrogen bonded to the carboxyl­ate O atoms of the anion *via* a pair of N—H⋯O hydrogen bonds, forming an *R* ^2^_2_(8) ring motif. The ion pairs are connected *via* further N—H⋯O hydrogen bonds and a short C—H⋯O inter­action, forming layers lying parallel to the *bc* plane.

## Related literature

For background to pyrimidine compounds, see: Glidewell *et al.* (2003 ▶); Panneerselvam *et al.* (2004 ▶). For details of maleic acid, see: James & Williams (1974 ▶); Bertolasi *et al.* (1980 ▶). For hydrogen-bond motifs, see: Bernstein *et al.* (1995 ▶).
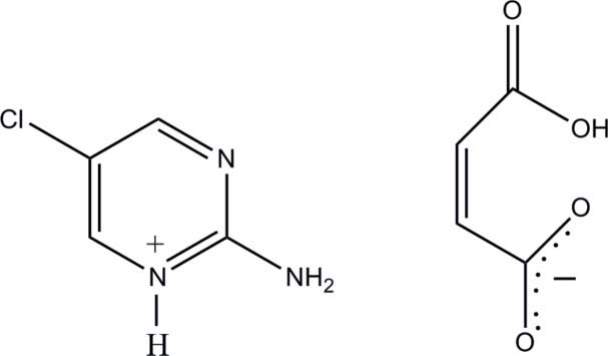


## Experimental

### 

#### Crystal data

C_4_H_5_ClN_3_^+^·C_4_H_3_O_4_^−^
*M* *_r_* = 245.62
Monoclinic, 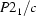

*a* = 9.3974 (6) Å
*b* = 5.5167 (4) Å
*c* = 20.0654 (13) Å
β = 95.264 (1)°
*V* = 1035.86 (12) Å^3^
*Z* = 4
Mo *K*α radiation
μ = 0.37 mm^−1^
*T* = 296 K
0.42 × 0.36 × 0.13 mm

#### Data collection

Bruker APEXII DUO CCD diffractometer
Absorption correction: multi-scan (*SADABS*; Bruker, 2009 ▶) *T*_min_ = 0.860, *T*_max_ = 0.954
12808 measured reflections
3443 independent reflections
2745 reflections with *I* > 2σ(*I*)
*R*_int_ = 0.023

#### Refinement

*R*[*F* ^2^ > 2σ(*F* ^2^)] = 0.038
*wR*(*F* ^2^) = 0.109
*S* = 1.04
3443 reflections
161 parameters
H atoms treated by a mixture of independent and constrained refinement
Δρ_max_ = 0.35 e Å^−3^
Δρ_min_ = −0.36 e Å^−3^

### 

Data collection: *APEX2* (Bruker, 2009 ▶); cell refinement: *SAINT* (Bruker, 2009 ▶); data reduction: *SAINT*; program(s) used to solve structure: *SHELXTL* (Sheldrick, 2008 ▶); program(s) used to refine structure: *SHELXTL*; molecular graphics: *SHELXTL*; software used to prepare material for publication: *SHELXTL* and *PLATON* (Spek, 2009 ▶).

## Supplementary Material

Crystal structure: contains datablock(s) global, I. DOI: 10.1107/S1600536811051646/hb6543sup1.cif

Structure factors: contains datablock(s) I. DOI: 10.1107/S1600536811051646/hb6543Isup2.hkl

Supplementary material file. DOI: 10.1107/S1600536811051646/hb6543Isup3.cml

Additional supplementary materials:  crystallographic information; 3D view; checkCIF report

## Figures and Tables

**Table 1:** Hydrogen-bond geometry (Å, °)

*D*—H⋯*A*	*D*—H	H⋯*A*	*D*⋯*A*	*D*—H⋯*A*
N2—H1*N*2⋯O4^i^	0.881 (19)	1.810 (18)	2.6897 (14)	177.6 (19)
N3—H1*N*3⋯O1^ii^	0.860 (17)	2.592 (18)	3.0814 (16)	117.2 (14)
N3—H1*N*3⋯O2^ii^	0.860 (17)	2.128 (17)	2.9795 (16)	170.2 (16)
N3—H2*N*3⋯O3^i^	0.893 (18)	1.975 (18)	2.8629 (17)	172.8 (16)
O1—H1*O*3⋯O3	0.86 (3)	1.60 (3)	2.4514 (15)	179 (3)
C2—H2*A*⋯O2^iii^	0.93	2.39	3.3117 (17)	173

Symmetry codes: (i) 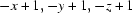; (ii) 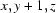; (iii) 
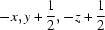
.

**Fig. 1.**
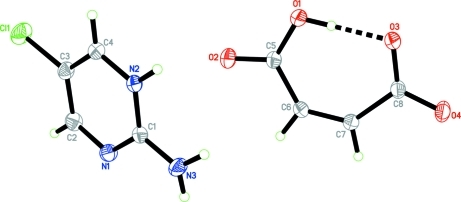
The asymmetric unit of the title compound, showing 50% probability displacement ellipsoids. Intramolecular hydrogen bonds shown by dashed lines.

**Fig. 2.**
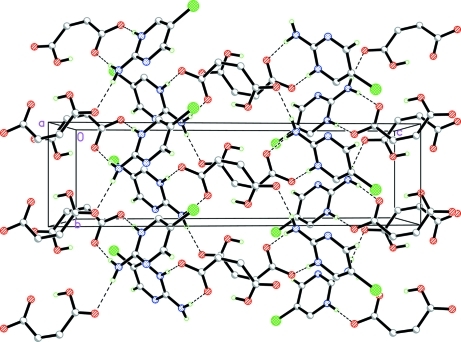
The crystal packing of title compound (I).

## supplementary crystallographic information

### Comment

Pyrimidine compounds have attracted much attention for their biological activities and molecular structures. The crystal structures of some 2-amino-substituted pyrimidine compounds, such as 2-amino-4-methoxy 6-methylpyrimidine (Glidewell *et al.*, 2003) and 2-amino-4,6-dimethyl pyrimidinium bromide (Panneerselvam *et al.*, 2004) have previously been elucidated.  A study of the structural chemistry of maleic acid and related substances arises from the fact that these systems possess short but highly strained hydrogen bonds (James & Williams, 1974). The crystal structures of maleic acid (James & Williams, 1974) and carbinoxamine maleate (Bertolasi *et al.*, 1980) have been reported in the literature. We report here the molecular structure of a title compound (I), formed from the reaction of 2-amino-5-chloropyrimidine with maleic acid. It was prepared in order to extend our study on D—H···A hydrogen bonding in organic systems.

The asymmetric unit of the title compound is shown in Fig. 1. The 2-amino-5-chloropyridinium (N1,N2/C1–C4) cation is essentially planar, with a maximum deviation of 0.004 (1) Å for atom N1.  In the 2-amino-5- chloropyrimidine molecule, a wide angle [C1—N2—C4 = 121.33 (10)°] is subtended at the protonated N2 atom.  In the hydrogen malate anion, an intramolecular O—H···O hydrogen bond generates an *S*(7) (Bernstein *et al.*, 1995) ring and results in a folded conformation.

In the crystal structure, (Fig. 2), the protonated N atom and the 2-amino group of the cation are hydrogen bonded to the carboxylate O atoms of the anion *via* a pair of N—H···O hydrogen bonds, forming an *R*^2^_2_(8) ring motif. The ion pairs are further connected *via* N—H···O and C—H···O hydrogen bonds (Table 1), forming a layer parallel to the *bc* plane.

### Experimental

A hot methanol solution (20 ml) of 2-amino-5-chloropyrimidine (32 mg, Aldrich) and maleic acid (29 mg, Merck) were mixed and warmed over a heating magnetic stirrer hotplate for a few minutes. The resulting solution was allowed to cool slowly at room temperature and colourless blocks of the title compound appeared after a few days.

### Refinement

Atoms H1N2, H1N3, H2N3 and H1O3 were located from a difference Fourier maps and refined freely [N–H = 0.858 (19)–0.89 (2) Å and O–H = 0.86 (3) Å]. The remaining H atoms were positioned geometrically [C–H = 0.93 Å] and were refined using a riding model, with *U*_iso_(H) = 1.2 *U*_eq_(C).

### Crystal data

**Table:**

C_4_H_5_ClN_3_^+^·C_4_H_3_O_4_^−^	*F*(000) = 504
*M**_r_* = 245.62	*D*_x_ = 1.575 Mg m^−^^3^
Monoclinic, *P*2_1_/*c*	Mo *K*α radiation, λ = 0.71073 Å
Hall symbol: -P 2ybc	Cell parameters from 4623 reflections
*a* = 9.3974 (6) Å	θ = 2.8–31.4°
*b* = 5.5167 (4) Å	µ = 0.37 mm^−^^1^
*c* = 20.0654 (13) Å	*T* = 296 K
β = 95.264 (1)°	Block, colourless
*V* = 1035.86 (12) Å^3^	0.42 × 0.36 × 0.13 mm
*Z* = 4	

### Data collection

**Table:**

Bruker APEXII DUO CCD diffractometer	3443 independent reflections
Radiation source: fine-focus sealed tube	2745 reflections with *I* > 2σ(*I*)
graphite	*R*_int_ = 0.023
φ and ω scans	θ_max_ = 31.7°, θ_min_ = 2.0°
Absorption correction: multi-scan (*SADABS*; Bruker, 2009)	*h* = −13→13
*T*_min_ = 0.860, *T*_max_ = 0.954	*k* = −7→8
12808 measured reflections	*l* = −29→29

### Refinement

**Table:**

Refinement on *F*^2^	Primary atom site location: structure-invariant direct methods
Least-squares matrix: full	Secondary atom site location: difference Fourier map
*R*[*F*^2^ > 2σ(*F*^2^)] = 0.038	Hydrogen site location: inferred from neighbouring sites
*wR*(*F*^2^) = 0.109	H atoms treated by a mixture of independent and constrained refinement
*S* = 1.04	*w* = 1/[σ^2^(*F*_o_^2^) + (0.0515*P*)^2^ + 0.2504*P*] where *P* = (*F*_o_^2^ + 2*F*_c_^2^)/3
3443 reflections	(Δ/σ)_max_ = 0.001
161 parameters	Δρ_max_ = 0.35 e Å^−^^3^
0 restraints	Δρ_min_ = −0.36 e Å^−^^3^

### Special details

**Table:**

Geometry. All s.u.'s (except the s.u. in the dihedral angle between two l.s. planes) are estimated using the full covariance matrix. The cell s.u.'s are taken into account individually in the estimation of s.u.'s in distances, angles and torsion angles; correlations between s.u.'s in cell parameters are only used when they are defined by crystal symmetry. An approximate (isotropic) treatment of cell s.u.'s is used for estimating s.u.'s involving l.s. planes.
Refinement. Refinement of F^2^ against ALL reflections. The weighted R-factor wR and goodness of fit S are based on F^2^, conventional R-factors R are based on F, with F set to zero for negative F^2^. The threshold expression of F^2^ > 2σ(F^2^) is used only for calculating R-factors(gt) etc. and is not relevant to the choice of reflections for refinement. R-factors based on F^2^ are statistically about twice as large as those based on F, and R- factors based on ALL data will be even larger.

### Fractional atomic coordinates and isotropic or equivalent isotropic displacement parameters (Å^2^)

**Table:**

	*x*	*y*	*z*	*U*_iso_*/*U*_eq_	
Cl1	0.07132 (4)	0.34821 (8)	0.119864 (19)	0.05434 (13)	
O1	0.34314 (14)	0.1959 (2)	0.47054 (5)	0.0547 (3)	
O2	0.20761 (12)	0.3832 (2)	0.39206 (5)	0.0483 (3)	
O3	0.44057 (12)	0.28155 (19)	0.58566 (5)	0.0477 (3)	
O4	0.43276 (11)	0.5756 (2)	0.65981 (4)	0.0456 (2)	
N1	0.14846 (11)	0.8848 (2)	0.24914 (5)	0.0364 (2)	
N2	0.32969 (11)	0.59171 (19)	0.27016 (5)	0.0305 (2)	
N3	0.32863 (14)	0.9408 (2)	0.33347 (6)	0.0424 (3)	
C1	0.26868 (12)	0.8052 (2)	0.28452 (5)	0.0307 (2)	
C2	0.09165 (13)	0.7456 (3)	0.20036 (6)	0.0372 (3)	
H2A	0.0086	0.7977	0.1757	0.045*	
C3	0.15083 (13)	0.5224 (2)	0.18395 (6)	0.0343 (2)	
C4	0.27274 (13)	0.4492 (2)	0.22009 (6)	0.0335 (2)	
H4A	0.3160	0.3033	0.2104	0.040*	
C5	0.26812 (14)	0.3825 (2)	0.44879 (6)	0.0351 (3)	
C6	0.25815 (15)	0.6000 (2)	0.49162 (6)	0.0391 (3)	
H6A	0.2049	0.7265	0.4714	0.047*	
C7	0.31273 (15)	0.6453 (2)	0.55439 (6)	0.0387 (3)	
H7A	0.2921	0.7985	0.5703	0.046*	
C8	0.40123 (13)	0.4898 (2)	0.60285 (5)	0.0328 (2)	
H1N2	0.4062 (19)	0.537 (4)	0.2941 (9)	0.048 (5)*	
H1N3	0.2848 (19)	1.066 (3)	0.3469 (9)	0.047 (5)*	
H2N3	0.402 (2)	0.884 (3)	0.3604 (9)	0.051 (5)*	
H1O3	0.376 (3)	0.225 (5)	0.5110 (14)	0.093 (8)*	

### Atomic displacement parameters (Å^2^)

**Table:**

	*U*^11^	*U*^22^	*U*^33^	*U*^12^	*U*^13^	*U*^23^
Cl1	0.0550 (2)	0.0562 (2)	0.0486 (2)	−0.00891 (17)	−0.01285 (15)	−0.01739 (16)
O1	0.0821 (8)	0.0392 (6)	0.0379 (5)	0.0180 (5)	−0.0202 (5)	−0.0097 (4)
O2	0.0577 (6)	0.0494 (6)	0.0343 (5)	0.0033 (5)	−0.0154 (4)	−0.0049 (4)
O3	0.0627 (6)	0.0412 (5)	0.0358 (4)	0.0183 (5)	−0.0148 (4)	−0.0045 (4)
O4	0.0498 (5)	0.0551 (6)	0.0298 (4)	0.0150 (5)	−0.0085 (4)	−0.0093 (4)
N1	0.0357 (5)	0.0351 (5)	0.0367 (5)	0.0036 (4)	−0.0055 (4)	−0.0018 (4)
N2	0.0331 (5)	0.0313 (5)	0.0261 (4)	0.0025 (4)	−0.0023 (3)	0.0008 (4)
N3	0.0491 (7)	0.0373 (6)	0.0376 (5)	0.0081 (5)	−0.0136 (5)	−0.0095 (5)
C1	0.0338 (5)	0.0305 (5)	0.0272 (5)	0.0001 (4)	−0.0009 (4)	0.0010 (4)
C2	0.0319 (5)	0.0412 (7)	0.0369 (6)	−0.0007 (5)	−0.0065 (4)	0.0009 (5)
C3	0.0354 (6)	0.0361 (6)	0.0306 (5)	−0.0068 (5)	−0.0023 (4)	−0.0029 (5)
C4	0.0387 (6)	0.0310 (6)	0.0306 (5)	−0.0009 (5)	0.0018 (4)	−0.0018 (4)
C5	0.0399 (6)	0.0342 (6)	0.0298 (5)	−0.0010 (5)	−0.0045 (4)	−0.0006 (4)
C6	0.0492 (7)	0.0335 (6)	0.0326 (5)	0.0106 (5)	−0.0070 (5)	0.0002 (5)
C7	0.0495 (7)	0.0335 (6)	0.0316 (5)	0.0110 (5)	−0.0039 (5)	−0.0031 (5)
C8	0.0330 (5)	0.0381 (6)	0.0265 (5)	0.0040 (5)	−0.0012 (4)	−0.0004 (4)

### Geometric parameters (Å, °)

**Table:**

Cl1—C3	1.7201 (12)	N3—H1N3	0.858 (19)
O1—C5	1.3005 (16)	N3—H2N3	0.89 (2)
O1—H1O3	0.86 (3)	C2—C3	1.4027 (19)
O2—C5	1.2248 (15)	C2—H2A	0.9300
O3—C8	1.2645 (16)	C3—C4	1.3599 (17)
O4—C8	1.2475 (14)	C4—H4A	0.9300
N1—C2	1.3178 (17)	C5—C6	1.4837 (18)
N1—C1	1.3512 (15)	C6—C7	1.3393 (17)
N2—C4	1.3473 (15)	C6—H6A	0.9300
N2—C1	1.3527 (15)	C7—C8	1.4916 (17)
N2—H1N2	0.880 (18)	C7—H7A	0.9300
N3—C1	1.3192 (16)		
			
C5—O1—H1O3	108.0 (18)	C2—C3—Cl1	120.76 (9)
C2—N1—C1	117.60 (11)	N2—C4—C3	118.81 (11)
C4—N2—C1	121.33 (10)	N2—C4—H4A	120.6
C4—N2—H1N2	117.2 (12)	C3—C4—H4A	120.6
C1—N2—H1N2	121.4 (12)	O2—C5—O1	120.36 (12)
C1—N3—H1N3	120.2 (12)	O2—C5—C6	119.16 (12)
C1—N3—H2N3	120.3 (12)	O1—C5—C6	120.46 (11)
H1N3—N3—H2N3	117.3 (16)	C7—C6—C5	131.05 (12)
N3—C1—N1	119.11 (11)	C7—C6—H6A	114.5
N3—C1—N2	119.47 (11)	C5—C6—H6A	114.5
N1—C1—N2	121.41 (10)	C6—C7—C8	130.40 (12)
N1—C2—C3	122.92 (11)	C6—C7—H7A	114.8
N1—C2—H2A	118.5	C8—C7—H7A	114.8
C3—C2—H2A	118.5	O4—C8—O3	122.95 (11)
C4—C3—C2	117.92 (11)	O4—C8—C7	116.77 (11)
C4—C3—Cl1	121.32 (10)	O3—C8—C7	120.28 (10)
			
C2—N1—C1—N3	179.67 (12)	C2—C3—C4—N2	0.81 (18)
C2—N1—C1—N2	0.63 (18)	Cl1—C3—C4—N2	−179.38 (9)
C4—N2—C1—N3	−179.27 (12)	O2—C5—C6—C7	179.72 (16)
C4—N2—C1—N1	−0.23 (18)	O1—C5—C6—C7	−2.0 (2)
C1—N1—C2—C3	−0.3 (2)	C5—C6—C7—C8	−0.8 (3)
N1—C2—C3—C4	−0.4 (2)	C6—C7—C8—O4	−177.01 (16)
N1—C2—C3—Cl1	179.77 (10)	C6—C7—C8—O3	2.8 (2)
C1—N2—C4—C3	−0.52 (17)		

### Hydrogen-bond geometry (Å, °)

**Table:**

*D*—H···*A*	*D*—H	H···*A*	*D*···*A*	*D*—H···*A*
N2—H1N2···O4^i^	0.881 (19)	1.810 (18)	2.6897 (14)	177.6 (19)
N3—H1N3···O1^ii^	0.860 (17)	2.592 (18)	3.0814 (16)	117.2 (14)
N3—H1N3···O2^ii^	0.860 (17)	2.128 (17)	2.9795 (16)	170.2 (16)
N3—H2N3···O3^i^	0.893 (18)	1.975 (18)	2.8629 (17)	172.8 (16)
O1—H1O3···O3	0.86 (3)	1.60 (3)	2.4514 (15)	179 (3)
C2—H2A···O2^iii^	0.93	2.39	3.3117 (17)	173

Symmetry codes: (i) −*x*+1, −*y*+1, −*z*+1; (ii) *x*, *y*+1, *z*; (iii) −*x*, *y*+1/2, −*z*+1/2.
